# Unravelling mummies: cryptic diversity, host specificity, trophic and coevolutionary interactions in psyllid – parasitoid food webs

**DOI:** 10.1186/s12862-017-0959-2

**Published:** 2017-06-06

**Authors:** Aidan A. G. Hall, Martin J. Steinbauer, Gary S. Taylor, Scott N. Johnson, James M. Cook, Markus Riegler

**Affiliations:** 1 Hawkesbury Institute for the Environment, Western Sydney University, Locked Bag 1797, Penrith, NSW 2751 Australia; 2 Department of Ecology, Environment & Evolution, La Trobe University, Melbourne, VIC 3086 Australia; 3 Australian Centre for Evolutionary Biology and Biodiversity, School of Biological Sciences, The University of Adelaide, Adelaide, SA 5005 Australia

**Keywords:** Coevolution, Conserved trophic roles, Cryptic species, Diversification, Heteronomous hyperparasitoid, Parasitoid

## Abstract

**Background:**

Parasitoids are hyperdiverse and can contain morphologically and functionally cryptic species, making them challenging to study. Parasitoid speciation can arise from specialisation on niches or diverging hosts. However, which process dominates is unclear because cospeciation across multiple parasitoid and host species has rarely been tested. Host specificity and trophic interactions of the parasitoids of psyllids (Hemiptera) remain mostly unknown, but these factors are fundamentally important for understanding of species diversity, and have important applied implications for biological control.

**Results:**

We sampled diverse parasitoid communities from eight *Eucalyptus-*feeding psyllid species in the genera *Cardiaspina* and *Spondyliaspis*, and characterised their phylogenetic and trophic relationships using a novel approach that forensically linked emerging parasitoids with the presence of their DNA in post-emergence insect mummies. We also tested whether parasitoids have cospeciated with their psyllid hosts. The parasitoid communities included three *Psyllaephagus* morphospecies (two primary and, unexpectedly, one heteronomous hyperparasitoid that uses different host species for male and female development), and the hyperparasitoid, *Coccidoctonus psyllae*. However, the number of genetically delimited *Psyllaephagus* species was three times higher than the number of recognisable morphospecies, while the hyperparasitoid formed a single generalist species. In spite of this, cophylogenetic analysis revealed unprecedented codivergence of this hyperparasitoid with its primary parasitoid host, suggesting that this single hyperparasitoid species is possibly diverging into host-specific species. Overall, parasitoid and hyperparasitoid diversification was characterised by functional conservation of morphospecies, high host specificity and some host switching between sympatric psyllid hosts.

**Conclusions:**

We conclude that host specialisation, host codivergence and host switching are important factors driving the species diversity of endoparasitoid communities of specialist host herbivores. Specialisation in parasitoids can also result in heteronomous life histories that may be more common than appreciated. A host generalist strategy may be rare in endoparasitoids of specialist herbivores despite the high conservation of morphology and trophic roles, and endoparasitoid species richness is likely to be much higher than previously estimated. This also implies that the success of biological control requires detailed investigation to enable accurate identification of parasitoid-host interactions before candidate parasitoid species are selected as biological control agents for target pests.

**Electronic supplementary material:**

The online version of this article (doi:10.1186/s12862-017-0959-2) contains supplementary material, which is available to authorized users.

## Background

Most insects suffer attack by parasitoids that can influence host population dynamics [1]. Parasitoids are hyperdiverse in their membership to different trophic levels (e.g. primary parasitoids or hyperparasitoids) and in species richness. In fact, it is estimated that hymenopteran parasitoids alone make up more than 20% of the world’s insect species, yet only about 1% of all parasitoid species have been described [1, 2].

The predominant drivers of parasitoid diversification are niche and host specificity [3–6]. In niche specific parasitism, hosts that share ecological niches such as location, feeding niche or host plant, may share very similar parasitoid communities [7]. In contrast, host specific parasitism relates to parasitoids having a single host species or host species that are phylogenetically closely related with each other [8]. Assessing parasitoid diversification can be a difficult task as it requires knowledge of the species identity and ecology of both the host and parasitoid across multiple systems [9].

Cophylogenetic analysis can provide insights into speciation and diversification if the ecology of the host species and their respective parasitoids is known. However, very few studies have tested for codivergence between insect hosts and their parasitoids (but see [10–14]), and we are aware of none that have investigated cophylogenetic relationships between hosts, primary parasitoids and their hyperparasitoids. Given that hyperparasitoids can be host specific to either the host herbivore (e.g. [15]) or the primary parasitoid (e.g. [16]), such coevolutionary relationships can be predicted, and cophylogenetic analyses should provide further insight into this.

Studying host – parasitoid food webs can be very challenging due to the undescribed diversity of parasitoids [17]. DNA barcoding and molecular approaches have greatly improved sensitivity and accuracy in species identification and food web studies [18–20]. For example, DNA barcoding has sometimes demonstrated that parasitoid species thought to be generalists are in fact host specific cryptic species, and hence parasitoid species richness is likely to be far higher than currently estimated [21, 22].

*Eucalyptus*-feeding psyllids, native to Australia, are highly diverse and host specific, with host ranges of either a single or several *Eucalyptus* species [23]. They can cause significant damage to trees during heavy infestations [24], and have become serious invasive pests of *Eucalyptus* plantations overseas [25–27]. The principal natural enemies of psyllids are chalcidoid parasitoids (Hymenoptera), including species from the families Aphelinidae, Encyrtidae, Eulophidae and Pteromalidae [28]. Of particular significance are species belonging to the encyrtid genus *Psyllaephagus* Ashmead. This genus includes morphologically difficult to distinguish species, and both primary parasitoids and hyperparasitoids have been described [28]. So far, around 110 *Psyllaephagus* species are known in Australia [29]; however, it is estimated that the actual diversity in Australia might be as high as 1000 species [30]. The dearth of knowledge about host specificity and trophic roles of *Psyllaephagus* spp. hinders assessment of their capacity to regulate psyllid abundance [31], and the implementation of biological control programs against invasive psyllids [32, 33]. *Psyllaephagus* species are solitary koinobiont endoparasitoids [29], that is they feed inside still developing hosts before eventually killing them with the emergence of only one parasitoid from a parasitised host. It is expected that koinobiont endoparasitoids generally have greater host specificity because the parasitoids are constrained by their intimate physiological relationship with their hosts [7].

*Cardiaspina* Crawford (Hemiptera: Aphalaridae) includes the most damaging of *Eucalyptus*-feeding psyllids [24, 34]. Serious outbreaks of several *Cardiaspina* species have occurred in mainland Australia [35–39]. Furthermore, *Cardiaspina fiscella* has spread to New Zealand [40] and Norfolk Island [41]. Previous research of parasitoids and hyperparasitoids attacking *Cardiaspina* psyllids leads to two contrasting yet untested hypotheses. There are either (1) several morphologically distinct parasitoid species with a broader psyllid host range on *Eucalyptus*, suggesting niche specific parasitism, or, conversely (2) highly host specific parasitisation with different sets of parasitoid species for each *Cardiaspina* species. Given that *Cardiaspina* psyllids are known *Eucalyptus* specialists that have diversified only on this host plant genus [23], it is possible to hypothesise further that parasitoid communities associated with *Cardiaspina* have either cospeciated with diverging psyllid host lineages specialised to different *Eucalyptus*, or, alternatively, codifferentiated with psyllid species, i.e. with diverging psyllid host lineages providing new colonisation opportunities for different sets of parasitoid species.

We aimed to investigate the relative contribution of cospeciation and host switching to parasitoid community composition across several psyllid species that are closely and more distantly related to each other. Therefore, we examined parasitoid communities of psyllid species with similar ecological niches on different *Eucalyptus* species with partially overlapping geographic distributions. More specifically, we sampled parasitoid communities associated with seven species of *Cardiaspina* and one species of the closely related genus *Spondyliaspis* from different species of *Eucalyptus* across a wide geographic range in south eastern Australia. Given the limited ecological and taxonomic information available for this host – parasitoid system, we combined DNA characterisation of emerging parasitoid wasps and morphospecies-specific multiplex PCR on post-emergence mummies (parasitised psyllid nymphs) as a forensic DNA-based profiling approach to characterise parasitoid diversity, host specificity and trophic roles across several related psyllid species for the first time. We then performed multilocus cophylogenetic analyses to test for codivergence and host switches between hosts and their parasitoids. We hypothesised that some koinobiont endoparasitoid and hyperparasitoid species codiverge with their host species, and therefore, we predicted higher parasitoid than psyllid diversity, and higher parasitoid host specificity than anticipated from current knowledge about morphological diversity of this parasitoid genus. We also expected that parasitoid communities may be impacted by the process of codifferentiation with some parasitoids shifting host preference as a consequence of the mosaic distribution of *Eucalyptus* species (e.g. [42]). Furthermore, we predicted that, based on expected cryptic diversity, a more generalist strategy of parasitism in parasitoids would be rare for parasitoids of specialist herbivores, even between phylogenetically closely related host psyllids that occupy highly similar ecological niches. We also predicted that our DNA-based profiling approach of parasitoid interactions would reveal new life history strategies in diverse parasitoid communities as a consequence of host specialisation and competition.

## Methods

### Field sampling and identification of parasitoid morphotypes

Parasitoids were collected from populations of seven *Cardiaspina* species and one population of a *Spondyliaspis* species from sites in Sydney, New South Wales (NSW), in Canberra, Australian Capital Territory (ACT) and Keith south east of Adelaide, South Australia (SA) (Additional file 1: Table S1). According to adult and lerp (protective covers excreted by nymphs) morphology, host tree association and DNA characterisation, *Cardiaspina* populations were assigned to six species (*C. albitextura* Taylor, *C. densitexta* Taylor, *C. fiscella* Taylor, *C. maniformis* Taylor, *C. tenuitela* Taylor and *C. vittaformis* Froggatt) that represent a phylogenetically broad diversity [43] of 24 known species of the genus *Cardiaspina* [23]. *Cardiaspina* from *Eucalyptus moluccana* Roxb. (Grey Box) was not assigned to any described species due to its recently described *Eucalyptus* association (GB *Cardiaspina* sp.) and uncertain species status [35]. First morphological identification placed it near *C. densitexta* [34] but molecular evidence did not unequivocally support this and clustered this species with both *C. densitexta* and *C. tenuitela*, two species feeding on other box eucalypts [43]. For the *Spondyliaspis* sp. and five of the seven *Cardiaspina* species, host plant leaves infested with psyllids were collected from trees within arm’s reach from the ground and placed into zip-lock bags. Leaf collections were performed when psyllid nymphs were between third and fifth instar. The zip-lock bags containing the leaves were monitored daily for the development of mummies, which were then placed into individual gelatine capsules. Following emergence, parasitoids and post-emergence mummies were separated into tubes containing absolute ethanol for subsequent analysis. For the remaining two *Cardiaspina* species, *C. densitexta* and *C. vittaformis*, we did not obtain sufficient numbers of mummies to be placed into gelatine capsules, and therefore only adult parasitoid wasps were collected from zip-lock bags with infested leaves. Adult parasitoids were grouped into morphologically distinct morphotypes with males and females forming different morphotypes. Sex was determined based on ovipositor and antennal morphology [28, 44]. Later, sexes were grouped into morphospecies based on their molecular identity, and for some morphospecies only females were found. The translucent versus opaque (black) appearance of mummies was also informative for species differentiation. The previous assessment of the GB *Cardiaspina* sp. parasitoid community [35] revealed seven morphotypes (i.e. four female and three male morphotypes) that likely represented four morphospecies, three of which were sexually dimorphic and one that appeared to be only female. The parasitoid morphotypes from GB *Cardiaspina* sp. and *C. fiscella* were also confirmed by John La Salle and vouchered with the Australian National Insect Collection (CSIRO, Canberra) (Additional file 1: Table S2).

### DNA characterisation of parasitoid morphospecies

Parasitoids were assigned to eight morphotypes (four female and four male morphotypes) which were later grouped into four major morphospecies by matching DNA sequences of female and male morphotypes – two primary parasitoid (P1 and P2), one hyperparasitoid (H) and one heteronomous hyperparasitoid (HH) (i.e. female HH developed from parasitised psyllids while male HH emerged from parasitised P1), as well as one rare aphelinid morphospecies. Where possible, four adult wasp specimens per morphospecies from each psyllid species were DNA profiled. For morphotypes for which only few individuals were obtained, DNA was extracted non-destructively following the protocol described in King et al. [45], while for the other morphotypes DNA was extracted after tissue homogenisation. DNA characterisation was performed with mitochondrial cytochrome b (*cytb*) [CB1 and CB2; 46], and the D2 expansion segment of nuclear 28S rDNA (D2–3551 F and D2–4057 R; [46]). The reaction mix for both loci comprised 1 × GoTaq PCR buffer, 0.5 U GoTaq DNA polymerase (Promega, Madison, WI, USA), 3 mM MgCl_2_, 0.1 mM of each dNTP, 0.5 μM of each primer and 2 μL DNA template in a 20 μL reaction volume. The PCR conditions for *cytb* were 94 °C for 3 min, 30 cycles of 95 °C for 15 s, 45 °C for 20 s and 72 °C for 30 s, with a final extension for 10 min at 72 °C, and for 28S rDNA the PCR conditions were 94 °C for 3 min, 30 cycles of 94 °C for 45 s, 55 °C for 30 s and 72 °C for 90 s, with a final extension for 10 min at 72 °C using a BioRad DNA engine Dyad®, Peltier Thermal Cycler (CA, USA). PCR amplicons were sequenced by Macrogen (Korea), and DNA sequences were edited in Sequencher 4.10.1 (Gene Codes Corporation, Ann Arbor, MI, USA) and deposited in GenBank (Additional file 1: Table S3).

### Phylogenetic analysis and species delimitation

A phylogeny of the parasitoid morphospecies from each psyllid population was constructed based on concatenated *cytb* and 28S rDNA sequences. Sequence alignments were performed in Clustal X 2.1 [47]. Evolutionary models were selected for the two gene datasets separately using Bayesian Information Criterion in MEGA 6.06 [48]; HKY + G for *cytb* and T92 + G for 28S rDNA. Phylogenetic relationships were estimated for gene and concatenated trees (with highly similar topologies) using Bayesian inference implemented in MrBayes 3.2.2 [49]. Posterior probabilities were calculated using four independent chains, including one cold, for 5 million generations, sampling every 100 generations, or until convergence was reached (< 0.01). The first 25% of trees generated were discarded and a 50% majority rule consensus tree was returned. FigTree 1.4.0 [50] was used to view the trees. Putative species boundaries were found for the gene trees of both loci using the bPTP web server [51] and the default parameters. Sequence divergence for both loci was calculated using the p-distance analysis in MEGA.

A psyllid host phylogeny was constructed from concatenated sequence data of mitochondrial genes *COI* (506 bp) [52] and *cytb* (398 bp) [53], and nuclear genes *wg* (268 bp)*, EF-1 alpha* (281 bp) and *CAD* (323 bp) designed for this study (Additional file 1: Tables S4 and S5). Also included was *COI* sequenced from three *Spondyliaspis* sp. individuals (KT388061). Some gene sequences were not obtained for *Spondyliaspis* sp. and they were therefore coded as missing data in MrBayes 3.2.2. The psyllid gene tree topologies were highly similar and the concatenated tree provided greater resolution and was therefore deemed best for further analyses [54]. Putative species boundaries were found for the concatenated psyllid phylogeny using the bPTP web server.

### Identification of trophic interactions

A morphospecies-specific multiplex PCR on post-emergence mummies was used to examine trophic interactions. This involved the DNA sequence analysis of emerging parasitoids, the design of morphospecies-specific primers and morphospecies-specific PCR testing of post-emergence mummies. If DNA of the psyllid host and only the emerging parasitoid species was consistently detected in post-emergence mummies then this was indicative that the emerging parasitoid was a primary parasitoid. If DNA of the host and two parasitoid species was consistently present in post-emergence mummies, and the DNA of the emerged parasitoid species was not detected alone in any mummy (i.e. without the presence of any other parasitoid species found in this experiment), then the emerged species was determined to be a hyperparasitoid of the primary parasitoid species that did not emerge.

Morphospecies-specific primers were designed based on morphospecies alignments of *cytb* sequences using SP-Designer v7.0 [55]. Sequences of P1 and HH from *C. albitextura* and *C. tenuitela* were too different to P1 and HH from the other host species. Therefore their specific primers had to be designed from separate alignments. Parameters were set for each primer pair to give a minimum of two diagnostic sites within the last five nucleotide positions at the 3′ end of each primer, different amplicon lengths (145–400 bp) and similar annealing temperatures to allow for multiplexing (Additional file 1: Table S6). Amplicon sizes for the primers of P1 and HH from *C. albitextura* and *C. tenuitela* were not different enough to differentiate them from P2 and H. Therefore, for parasitoids of these two host species, the multiplex PCR had to be performed in two stages; one multiplex PCR for P1 and HH, and another for P2 and H. Morphospecies-specificity and ability of the primers to co-amplify in different template DNA combinations were confirmed by both single and multiplex PCR using adult wasp and psyllid DNA. DNA sequencing of PCR products confirmed that they were not artefacts.

DNA from mummies was extracted using the Gentra Puregene Tissue Kit (Qiagen, Germany) following the manufacturer’s instructions. Mummy extracts were deemed informative if DNA of both the emerging parasitoid and psyllid were detected by PCR. Psyllid DNA was amplified with primers for *wg*. For each parasitoid morphospecies and sex from each host population, two to 20 informative mummies were subjected to multiplex PCR in a 10 μL reaction comprising 1× Qiagen Multiplex PCR Master Mix, 0.2 μM morphospecies-specific forward and reverse primers, 1 μL DNA template and UltraPure water. Cycling conditions according to the Qiagen Multiplex PCR Kit protocol were used with 57 °C as annealing temperature.

A subset of the post-emergence mummy extracts was also tested with individual morphospecies-specific primer pairs to confirm the multiplex PCR results. This was to control for low DNA yield in post-emergence mummies, especially as the primary parasitoids could have been consumed by hyperparasitoids. All single and multiplex PCR reactions included a negative control, without a DNA template, and a positive control, containing an equal mix of all parasitoid species and psyllid DNA.

### Cophylogenetic analysis

Phylogenetic congruence (the degree of match between two phylogenies) may indicate parallel divergence of interacting species, and therefore can reveal patterns of speciation and diversification of species. Phylogenetic congruence was examined between all parasitoid morphospecies and their respective hosts, i.e. between 1) P1 and their psyllid hosts; 2) P2 and their psyllid hosts; 3) the hyperparasitoid, H, and its host (P2), as well as the respective psyllid hosts of P2; and 4) HH and its hosts (P1 and psyllids). Parasitoid phylogenies were constructed from the dominant genotype (GT) for each morphospecies, or GT1 if there was no dominant genotype (Additional file 1: Table S3). Both host and parasitoid phylogenies were rooted with the most distantly related species according to p-distances. As different approaches exist for cophylogenetic analyses [56], we employed three current methods that have recently also been successfully applied to test codivergence and host switching of bacterial endosymbionts and their psyllid hosts [43]: phylogenetic topology-based TreeMap 3 [57], and the two phylogenetic distance-based methods, ParaFit [58] and Hommola’s cospeciation test (hereafter HCT) [59]. Briefly, TreeMap 3 was used to reconstruct tanglegrams and to assess congruence between host and parasitoid phylogenies. Statistical significance was evaluated by mapping 1000 random parasitoid trees onto the host tree to estimate whether the number of codivergence events was due to chance alone. ParaFit and HCT were implemented in R 3.2.2 [60] using the ape 3.3 package [61]. Both of these methods were assessed from patristic distance matrices for the host and parasitoid phylogenies, as well as an association matrix of the host – parasitoid links. The observed correlation between matrices was compared to a null distribution of matrix correlations created through 10,000 permutations.

## Results

### Collection and molecular identification of parasitoid morphospecies

A total of 1023 parasitoid wasps emerged from mummies in gelatine capsules or were collected from zip-lock bags containing nymphs of eight species of *Cardiaspina* and *Spondyliaspis* (Table 1). Eight distinct parasitoid morphotypes (with separate female and male morphotypes) belonging to five morphospecies (female and male morphotypes were paired into morphospecies, while for some morphospecies only females were collected) were identified across all psyllid populations. According to existing identification keys and previous extensive population surveys of GB *Cardiaspina* sp. [35], three morphospecies (P1, P2 and HH) belonged to *Psyllaephagus* and the other (H) was *Coccidoctonus psyllae* Riek (Hymenoptera: Encyrtidae). Apart from these four major morphospecies, one additional rare species was found, as females only, from the *C. tenuitela* and *Spondyliaspis* sp. populations. The closest 28S rDNA BLAST match for this species was *Euryischia* sp. (Hymenoptera: Aphelinidae).

**Table 1:** Number of parasitoid wasps emerged from mummies isolated in individual gelatine capsules or from nymphs in zip-lock bags, separated by morphotype, sex and host species

Host	P1 (*Psyllaephagus* sp.)	P2 (*Psyllaephagus* sp.)		H (*Coccidoctonus psyllae*)		HH (*Psyllaephagus* sp.)		Unknown species (Aphelinidae)	Total
	female	male	female	male	female	male	female	female	
*C. albitextura*	67	2	10	17	50	0	10	0	156
*C. densitexta*	75	2	3	2	2	0	0	0	84
*C. fiscella*	1	2	2	59	60	4	9	0	137
*C. maniformis*	12	29	21	0	0	6	10	0	78
*C. tenuitela*	53	0	0	6	14	0	8	1	82
*C. vittaformis*	5	0	0	0	0	0	0	0	5
GB *Cardiaspina* sp.	301	57	27	26	26	10	9	0	456
*Spondyliaspis* sp.	0	4	9	3	0	2	5	2	25
Total	514	96	72	113	152	22	51	3	1023

The number of wasp individuals sampled varied between five from *C. vittaformis* to 456 from GB *Cardiaspina* sp. populations. P1 was the dominant morphospecies in five of the eight *Cardiaspina* populations, and was present in every *Cardiaspina* population (Table 1). P2 and H were found in six *Cardiaspina* populations, including in a few *Cardiaspina* populations where they were dominant or common. HH was less abundant, but still present in six *Cardiaspina* populations.

**Fig. 1:**
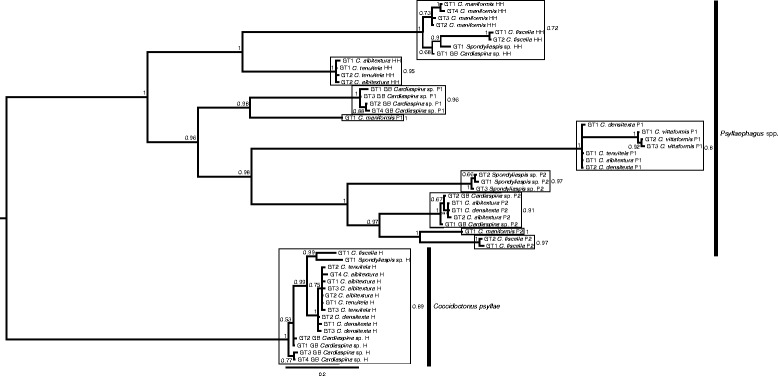
Majority consensus phylogeny of parasitoid species associated with seven *Cardiaspina* spp. and one *Spondyliaspis* sp. host populations. The phylogeny was constructed based on mitochondrial *cytb* (344 bp) and nuclear 28S rDNA (509 bp) using Bayesian inference. The genotype (GT) number, host species name and abbreviation of parasitoid morphospecies are included as tip labels. Abbreviations for parasitoid morphospecies are defined by their trophic roles, i.e. P1 and P2 (primary parasitoids), H (hyperparasitoid) and HH (heteronomous hyperparasitoid). P1, P2 and HH are *Psyllaephagus* species. The phylogeny was rooted with the H clade (*Coccidoctonus psyllae*). Bayesian posterior support values are provided at the nodes, and the scale bar represents the number of substitutions per site. Frames enclose bPTP putative species according to *cytb*, and the posterior delimitation probabilities are provided outside of the frames

DNA sequences were obtained for *cytb* (344 bp) and 28S rDNA (509 bp) of all parasitoid morphospecies from each host population (Additional file 1: Table S3), with a few exceptions: only a single P1 individual was obtained from the *C. fiscella* population and it failed to yield readable sequences; adult specimens of P2 from the *C. tenuitela* and P1 from the *Spondyliaspis* sp. populations were not obtained for DNA characterisation, but were detected by PCR in mummies; and the aphelinid only amplified for 28S rDNA from the *Spondyliaspis* sp. host while attempts to amplify *cytb* were unsuccessful. Within the concatenated *cytb* and 28S rDNA phylogeny, the morphospecies of HH, H and P2 all constituted monophyletic clades, while P1 was divided into two paraphyletic clades (Fig. 1).

For *cytb*, P1 from *C. albitextura*, *C. densitexta*, *C. tenuitela* and *C. vittaformis* were ≤2.6% diverged and were deemed to be a single species according to bPTP (Table 2, Fig. 1). P1 from *C. maniformis* and GB *Cardiaspina* sp. were highly diverged (13.1%) from each other and from P1 of the other host species (17.2–18.6%), and were, according to bPTP, separate species. P2 from *C. albitextura*, *C. densitexta* and GB *Cardiaspina* sp. were 0.3% diverged from each other and possibly a single species. P2 from *C. fiscella*, *C. maniformis* and *Spondyliaspis* sp. were more diverged from each other and from the other P2 (8.4–16.3%), and potentially represented three additional P2 species. HH from *C. albitextura* and *C. tenuitela* were <1% diverged from each other, but >15% diverged from HH of *C. fiscella*, *C. maniformis*, GB *Cardiaspina* sp. and *Spondyliaspis* sp., which were ≤4.7% diverged from each other; therefore, HH potentially formed two species. At <5% divergence between all populations, H, according to bPTP, constituted a single species. There was very little divergence between haplotypes within all morphospecies collected from the same host population, indicating an absence of cryptic species within host populations.

**Table 2:** Maximum *cytb* (344 bp) sequence divergence (%) comparisons of parasitoid morphospecies (P1, P2, H and HH) between populations of psyllid hosts

Comparison	P1	P2	H	HH
*C. albitextura* - *C. albitextura*	0	0.3	0.9	0.9
*C. albitextura - C. densitexta*	0.3	0.3	1.2	–
*C. albitextura - C. fiscella*	–	10.5	2.3	17.2
*C. albitextura - C. maniformis*	17.4	11.6	–	15.1
*C. albitextura - C. tenuitela*	0	–	0.6	0.9
*C. albitextura - C. vittaformis*	2.3	–	–	–
*C. albitextura -* GB *Cardiaspina* sp.	17.2	0.3	3.8	15.4
*C. albitextura - Spondyliaspis* sp.	–	14.8	3.5	16.9
*C. densitexta* - *C. densitexta*	0.3	0	0.6	–
*C. densitexta - C. fiscella*	–	10.5	1.7	–
*C. densitexta - C. maniformis*	17.4	11.6	–	–
*C. densitexta - C. tenuitela*	0.3	–	0.9	–
*C. densitexta - C. vittaformis*	2.6	–	–	–
*C. densitexta -* GB *Cardiaspina* sp.	17.2	0.3	3.2	–
*C. densitexta - Spondyliaspis* sp.	–	14.8	3.5	–
*C. fiscella* - *C. fiscella*	–	0.6	0	0.6
*C. fiscella - C. maniformis*	–	8.4	–	4.7
*C. fiscella - C. tenuitela*	–	–	2	17.4
*C. fiscella - C. vittaformis*	–	–	–	–
*C. fiscella -* GB *Cardiaspina* sp.	–	10.5	2	2.9
*C. fiscella - Spondyliaspis* sp.	–	14.8	2.9	3.2
*C. maniformis* - *C. maniformis*	0	0	–	0.6
*C. maniformis - C. tenuitela*	17.4	–	–	15.7
*C. maniformis - C. vittaformis*	18.6	–	–	–
*C. maniformis -* GB *Cardiaspina* sp.	13.1	11.6	–	1.7
*C. maniformis - Spondyliaspis* sp.	–	16.3	–	3.2
*C. tenuitela* - *C. tenuitela*	0	–	0.6	0.3
*C. tenuitela - C. vittaformis*	2.3	–	–	–
*C. tenuitela -* GB *Cardiaspina* sp.	17.2	–	3.5	15.7
*C. tenuitela - Spondyliaspis* sp.	–	–	3.2	17.2
*C. vittaformis* - *C. vittaformis*	0.6	–	–	–
*C. vittaformis -* GB *Cardiaspina* sp.	18.6	–	–	–
*C. vittaformis - Spondyliaspis* sp.	–	–	–	–
GB *Cardiaspina* sp. - GB *Cardiaspina* sp.	0.9	0.3	0.9	0
GB *Cardiaspina* sp. *- Spondyliaspis* sp.	–	14.8	4.9	2
*Spondyliaspis* sp. - *Spondyliaspis* sp.	–	0.6	0	0

28S rDNA sequence divergence patterns were similar to *cytb* yet smaller; P2, in particular, displayed very little divergence between host populations (≤ 2.3%) (Additional file 1: Table S7). Putative species delimitation of 28S rDNA was the same as for *cytb* except that P2 was suggested to be a single species, although with low support (0.57) (Additional file 1: Table S8). Furthermore, the support was generally higher for *cytb* species groupings than for 28S rDNA.

**Fig. 2:**
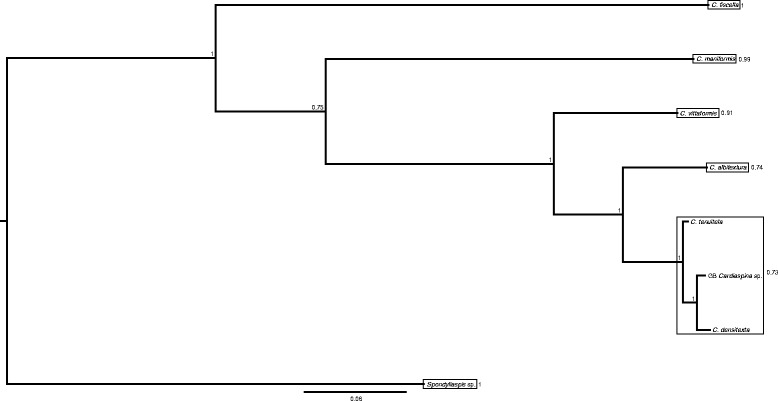
Majority consensus phylogeny of *Cardiaspina* and other psyllid species, constructed based on mitochondrial *COI* (506 bp) and *cytb* (398 bp), and nuclear *wg* (268 bp)*, EF-1 alpha* (281 bp) and *CAD* (323 bp) using Bayesian inference, and *Spondyliaspis* sp. to root the tree. Bayesian posterior support values are provided at the nodes, and the scale bar represents the number of substitutions per site. Frames enclose bPTP putative species, and the posterior delimitation probabilities are provided outside of the frames

The psyllid phylogeny separated the seven *Cardiaspina* species according to the described species, although *C. densitexta*, *C. tenuitela* and GB *Cardiaspina* sp. formed one clade that was indicated to constitute a single species by bPTP analysis (Fig. 2).

### Morphological characteristics and trophic interactions of parasitoids

Four morphospecies emerged from the mummies in gelatine capsules. H (*Co. psyllae*) was distinct from the other morphotypes (*Psyllaephagus* spp.) in the more setose male antennae, longer female ovipositor and lack of metallic green colour. P1 had a metallic green thorax and head, while P2 had a metallic green abdomen, which also differed in shape from P1. For P1 only females were found while the other morphospecies had both females and males, with species characteristic antennae in males. HH females were indistinguishable from P1 females but had a distinct genotype that matched the distinctive HH males; they also emerged from opaque mummies, whereas P1 emerged from translucent mummies.

The morphospecies-specific PCR on post-emergence mummies demonstrated that the function of the major parasitoid morphospecies was highly conserved across host systems (Table 3). Two of the four major morphospecies were primary parasitoids (P1 and P2), one was a hyperparasitoid (H) that attacked P2, and another was a heteronomous hyperparasitoid (HH) that used psyllids as host for female wasps and P1 as host for male wasps. It was not possible to establish the trophic level of the aphelinid species due to the low sample number. Besides emerged adult wasps from mummies (Table 1), we also found the DNA of P2 in H-hyperparasitised mummies in the *C. tenuitela* population, and the DNA of P1 in hyperparasitised mummies from which male HH emerged in the *Spondyliaspis* sp. population, indicating that these morphospecies were present in these host systems although we did not collect any emerging adult specimens from these host populations. No larval competition between the two primary parasitoid species was detected, because DNA of both P1 and P2 was never detected within the same mummy.

**Table 3:** Results of morphospecies-specific multiplex PCR screening of post-emergence *Cardiaspina* and *Spondyliaspis* mummies

Host species	Emerged parasitoid species	Emerged species DNA	Psyllid DNA	Other species DNA	Informative mummies	Uninformative mummies	Detection success (%)
*C. albitextura*	P1	Yes	Yes	No	20	40	33
	P2 female	Yes	Yes	No	7	3	70
	P2 male	Yes	Yes	No	2	0	100
	H female	Yes	Yes	P2	20	5	80
	H male	Yes	Yes	P2	15	2	88
	HH female	Yes	Yes	No	5	5	50
	HH male	–	–	–	–	–	–
*C. fiscella*	P1	Yes	Yes	No	0	1	0
	P2 female	Yes	Yes	No	2	0	100
	P2 male	Yes	Yes	No	2	0	100
	H female	Yes	Yes	P2	20	7	74
	H male	Yes	Yes	P2	20	6	77
	HH female	Yes	Yes	No	8	1	89
	HH male	Yes	Yes	P1	4	0	100
*C. maniformis*	P1	Yes	Yes	No	2	10	17
	P2 female	Yes	Yes	P2	17	4	81
	P2 male	Yes	Yes	P2	20	3	87
	H female	–	–	–	–	–	–
	H male	–	–	–	–	–	–
	HH female	Yes	Yes	No	8	2	80
	HH male	Yes	Yes	P1	4	2	67
*C. tenuitela*	P1	Yes	Yes	No	8	45	15
	P2 female	–	–	–	–	–	–
	P2 male	–	–	–	–	–	–
	H female	Yes	Yes	P2	14	0	100
	H male	Yes	Yes	P2	4	2	67
	HH female	Yes	Yes	No	5	3	63
	HH male	–	–	–	–	–	–
GB *Cardiaspina* sp.	P1	Yes	Yes	No	20	162	11
	P2 female	Yes	Yes	No	20	5	80
	P2 male	Yes	Yes	No	20	4	83
	H female	Yes	Yes	P2	20	6	77
	H male	Yes	Yes	P2	20	6	77
	HH female	Yes	Yes	No	8	1	89
	HH male	Yes	Yes	P1	3	7	30
*Spondyliaspis* sp.	P1	–	–	–	–	–	–
	P2 female	Yes	Yes	No	3	6	33
	P2 male	Yes	Yes	No	4	0	100
	H female	–	–	–	–	–	–
	H male	Yes	Yes	P2	2	1	67
	HH female	Yes	Yes	No	4	1	80
	HH male	Yes	Yes	P1	2	0	100

Uninformative mummies were PCR negative for either the psyllid host or the emerging parasitoid. For P1, only a female morphotype was found across all host species. Similarly for HH of *C. albitextura* and *C. tenuitela*, only a female morphotype was found. It was therefore concluded that P1 for all host species and HH from *C. albitextura* and *C. tenuitela* were thelytokous. HH from *C. albitextura* and *C. tenuitela* is likely a cryptic species that is distinct from other HH morphospecies

Overall, DNA detection success rate was very high, except for P1 which had a low detection success rate (Table 3). If the hyperparasitoid and psyllid host DNA were successfully detected in hyperparasitised mummies, then the primary parasitoid was also detected on every occasion.

### Cophylogenetic analysis

**Fig. 3:**
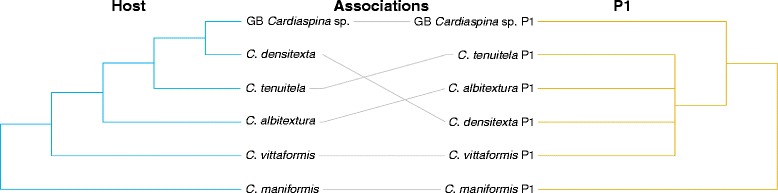
Tanglegram of concatenated host psyllid *COI* (506 bp), *cytb* (398 bp), *wg* (268 bp)*, EF-1 alpha* (281 bp) and *CAD* (323 bp), and concatenated primary parasitoid P1 *cytb* (344 bp) and 28S rDNA (509 bp) phylogenies, listed by their host associations

Phylogenetic congruence between psyllid hosts and P1 was not supported. TreeMap mapped the parasitoid phylogeny onto the host phylogeny and reconciled their evolutionary history through four possible types of evolutionary events: codivergence in host and parasitoid; duplication (divergence in parasitoid but not in host); lineage sorting/loss (divergence in host but not in parasitoid, or loss of parasitoid); host switching. TreeMap found five optimal solutions with a maximum of 6 codivergence events, 1–4 duplications, 0–10 lineage sorting/losses and 0–4 host switches by mapping P1 onto their psyllid hosts (Fig. 3). A statistical test could not be performed due to the P1 phylogeny not being fully resolved. The distance-based analyses found no congruence between the host and P1 phylogenies (ParaFitGlobal = 0.046, *P* = 0.101, ParaFit; *P* = 0.196, HCT).

**Fig. 4:**
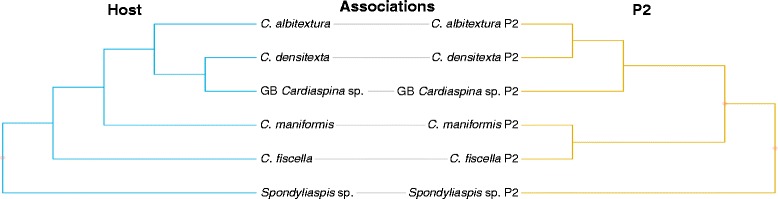
Tanglegram of concatenated host psyllid *COI* (506 bp), *cytb* (398 bp), *wg* (268 bp)*, EF-1 alpha* (281 bp) and *CAD* (323 bp), and concatenated primary parasitoid P2 *cytb* (344 bp) and 28S rDNA (509 bp) phylogenies, listed by their host associations. Dots indicate significance of congruence on individual nodes between host and parasitoid phylogenies

TreeMap did not find phylogenetic congruence for psyllid hosts and P2 (*P* = 0.73) based on just one optimal solution, with 6 codivergence events, 4 duplications, 6 lineage sorting/losses and 0 host switches (Fig. 4). However, both ParaFit (ParaFitGlobal = 0.004, *P* = 0.005) and HCT (*P* = 0.035) supported phylogenetic congruence between psyllid hosts and P2.

**Fig. 5:**
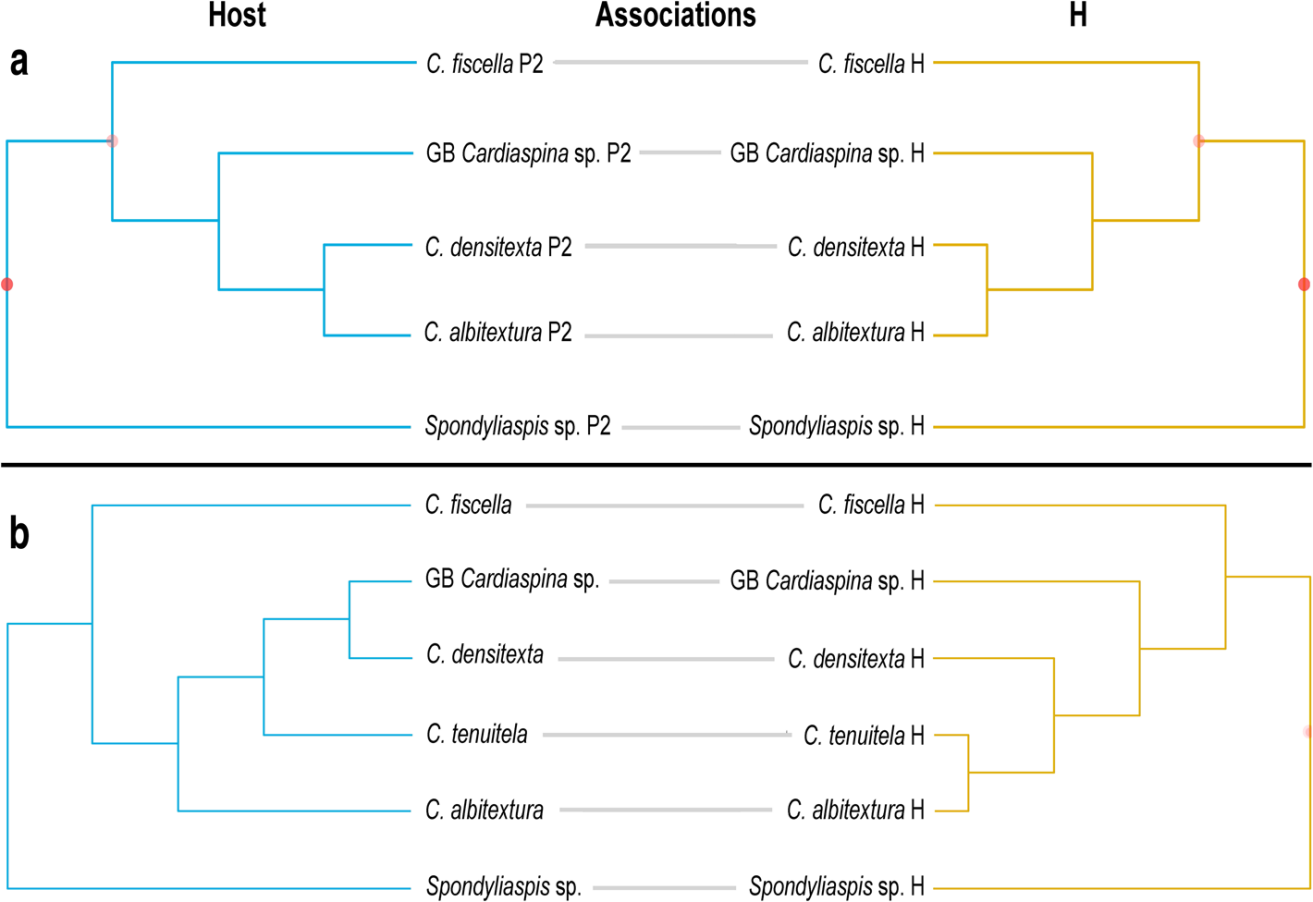
Tanglegram of (**a**) concatenated parasitoid P2 (host of H), and concatenated hyperparasitoid H *cytb* (344 bp) and 28S rDNA (509 bp) phylogenies, and (**b**) concatenated host psyllid *COI* (506 bp), *cytb* (398 bp), *wg* (268 bp)*, EF-1 alpha* (281 bp) and *CAD* (323 bp) and concatenated hyperparasitoid H *cytb* (344 bp) and 28S rDNA (509 bp) phylogenies, listed by their host associations. Dots indicate significance of congruence (darker shade represents higher significance) on individual nodes between host and parasitoid phylogenies

Phylogenetic congruence was supported between P2 and H based on ParaFit (ParaFitGlobal <0.001, *P* = 0.043), HCT (*P* = 0.016) and TreeMap (*P* = 0.01), which found just one optimal solution, 8 codivergence events and no other types of event (Fig. 5a). Furthermore the psyllids were also congruent with the phylogeny of H according to ParaFit (ParaFitGlobal <0.001, *P* = 0.02) and HCT (*P* = 0.015). However, TreeMap did not support phylogenetic congruence (*P* = 0.755), and found two solutions with a maximum of 6 codivergence events, 3–4 duplications, 7 lineage sorting/losses and 0–1 host switches (Fig. 5b).

**Fig. 6:**
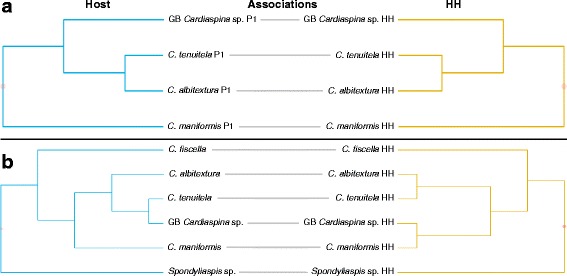
Tanglegram of (**a**) concatenated parasitoid P1 and concatenated heteronomous hyperparasitoid HH *cytb* (344 bp) and 28S rDNA (509 bp) phylogenies, and (**b**) concatenated host psyllid *COI* (506 bp), *cytb* (398 bp), *wg* (268 bp)*, EF-1 alpha* (281 bp) and *CAD* (323 bp) and concatenated heteronomous hyperparasitoid HH *cytb* (344 bp) and 28S rDNA (509 bp) phylogenies, listed by their host associations. Dots indicate significance of congruence (darker shade represents higher significance) on individual nodes between host and parasitoid phylogenies

Phylogenetic congruence between P1 and HH was supported by ParaFit (ParaFitGlobal <0.001, *P* = 0.043), and HCT (*P* = 0.084) and TreeMap (*P* = 0.07) were nearly significant. TreeMap found just one optimal solution with 6 codivergence events and no other events (Fig. 6a). The other hosts of HH, the psyllids, were not phylogenetically congruent with HH; ParaFit (ParaFitGlobal = 0.004, *P* = 0.205), HCT (*P* = 0.458) and TreeMap (*P* = 0.116), which found seven solutions with a maximum of 8 codivergence events, 2–4 duplications, 1–4 lineage sorting/losses and 0–5 host switches (Fig. 6b).

## Discussion

Our study revealed high genetic diversity in parasitoid communities associated with populations of eight psyllid species feeding on *Eucalyptus* trees. The populations comprised six recognised and phylogenetically diverse species of the psyllid genus *Cardiaspina*, as well as GB *Cardiaspina* sp. and one *Spondyliaspis* sp., and included significant defoliators of *Eucalyptus* in Australia and overseas. The sampled psyllid species are specific to one or few *Eucalyptus* species, and were sampled from either sympatric or geographically distant populations (maximum distance was close to 1400 km between Sydney and Adelaide). From our sampling we obtained four common parasitoid morphospecies with highly conserved trophic roles; two *Psyllaephagus* primary parasitoid morphospecies, one *Psyllaephagus* heteronomous hyperparasitoid morphospecies, and one obligate hyperparasitoid species, *Co. psyllae*. All three *Psyllaephagus* morphospecies contained cryptic genetic diversity that could represent up to nine cryptic species. Diversification was characterised by functional conservation of morphospecies (i.e. they were either primary parasitoid, hyperparasitoid or heteronomous hyperparasitoid across all host species), host specificity (to just one or few *Cardiaspina* species) and ecological niche specificity driven by geographical differentiation and *Eucalyptus* host tree distribution. These results suggest that even at a low host taxonomic scale, within a host psyllid genus specialised to *Eucalyptus* and diversified across *Eucalyptus* species, koinobiont endoparasitoids are highly restricted by their intimate relationships with their host species so that there may be little prospect of finding generalist species. This supports the view that host specialisation is a major factor in parasitoid speciation [3–6], and is also an important conclusion with respect to development of biological control programs for invasive species. Furthermore, by using our DNA-based profiling approach for host-parasitoid interactions we have discovered that one of the *Psyllaephagus* morphospecies is a heteronomous hyperparasitoid. Until now, heteronomous life histories (i.e. male and female individuals of a parasitoid species use a different host species) have only been described for species in another hymenopteran family (Aphelinidae) and one strepsipteran family (Myrmecolacidae) [62].

### Parasitoid morphospecies and trophic roles

The populations of eight psyllid species sampled across south eastern Australia represented one *Spondyliaspis* sp., six described *Cardiaspina* spp. and GB *Cardiaspina* sp. that has not been assigned to any known species due to its new host tree association [35]. However, DNA characterisation suggested that GB *Cardiaspina* sp., *C. densitexta* and *C. tenuitela* are either a single species or a complex of closely related sibling species [43]. The four major parasitoid morphospecies, consisting of three *Psyllaephagus* morphospecies and *Co. psyllae*, were present and abundant in most psyllid populations, including one *Psyllaephagus* morphospecies (P1) that was present in all psyllid populations.

The morphospecies-specific PCR approach on post-emergence mummies revealed highly conserved trophic roles for each morphospecies. Across all the five *Cardiaspina* and one *Spondyliaspis* species that we tested with our diagnostic method on post-emergence mummies, two primary parasitoid species, P1 and P2, one hyperparasitoid, H, and one heteronomous hyperparasitoid, HH, were detected.

With the exception of P1, detection of parasitoid DNA in mummies was high (> 60%). The poor detection success of P1 may be due to its apparent lack of a pupal sheath in the mummy [63], as also seen by the translucent appearance of mummies. Furthermore, in cases of hyperparasitism, DNA of the primary parasitoid host was always detected. We have demonstrated that our approach is very powerful for untangling host – endoparasitoid food webs, including in situations where there is no prior knowledge of any of the species involved. This confirmed theoretical predictions made in a previous study that used molecular diagnostics to detect DNA of a known parasitoid and a known hyperparasitoid species in post-emergence mummies of two aphid species [64]. Furthermore, our approach revealed heteronomous hyperparasitism for only the third time in insects; it has previously been detected in the hymenopteran family Aphelinidae and the strepsipteran family Myrmecolacidae [62]. Therefore this life history strategy may be more common than previously anticipated, but not readily detected due to the cryptic biology of many parasitoid species.

However, there are also limitations to our multiplex PCR approach; it cannot detect self- or conspecific superparasitisation where the same female, or a female of the same species, parasitises a host that is already parasitised. The design of target-specific primers will be difficult in systems with little genetic divergence between target species.

Our study is so far the best effort to characterise the genetic diversity and interactions within parasitoid communities of psyllids, and of *Cardiaspina* spp. specifically; P1 was confirmed to be present in populations of all eight host species, and it was the most abundant morphospecies in five. The genus *Psyllaephagus* is known for its marked sexual dimorphism [28]. P1, however, appeared to have only a female morphotype, thus we concluded that this morphospecies is probably thelytokous. In general, thelytoky appears to confer a fitness advantage over sexual reproduction because of the cost of producing males [65, 66]. However, it has also been suggested that asexual organisms accumulate deleterious mutations more quickly [67], and this places them at a disadvantage in the coevolutionary arms race with natural enemies [68]. P1 appeared resistant to the obligate hyperparasitoid, H, despite the co-occurrence of P1 and P2 (supporting high host specificity of H); the mechanisms behind this will require further investigation as P1 may prove to be a powerful biological control agent in host species outbreaks outside their native range (furthermore, some P1 species seem less host specific and can parasitise up to four *Cardiaspina* species). H appeared absent in the *C. maniformis* population in which P2 was abundant, therefore it is possible that P2 was able to outcompete P1 explaining the shift in this system to P2 as the dominant primary parasitoid. The dominance of H in the *C. fiscella* population and near complete suppression of its primary parasitoid host P1 could contribute to *C. fiscella* more frequently exhibiting outbreaks [24, 36, 39, 40].

We detected most parasitoid morphospecies even when only a single psyllid population per host species was sampled. However, not all major parasitoid morphospecies were recorded in psyllid populations for which we had a relatively low sampling effort. Irrespective of this, given that we have not detected any morphospecies that are specific to just one psyllid species, we are confident that we have sampled the key parasitoid species of the investigated hosts. Furthermore, while we only sampled one population per psyllid species, this limitation was counterbalanced by wide sampling of phylogenetically diverse representative species of this host genus across distances of about 1400 km in south eastern Australia, so that overall our study identified the major parasitoid and hyperparasitoid species in this host – parasitoid system across different regions and host species.

### Parasitoid cryptic species and diversification

Without our DNA-based profiling approach and phylogenetic characterisation, the primary parasitoids of *Cardiaspina* and *Spondyliaspis* would have appeared to consist of a few generalist morphospecies. However, genetic divergence across host populations of the three *Psyllaephagus* morphospecies indicated putative cryptic species: P1 could comprise three species (all of which appeared thelytokous); P2 four species, and HH two species, one from Sydney populations of *C. fiscella*, *C. maniformis*, GB *Cardiaspina* sp. and *Spondyliaspis* sp. that had both males and females, and a second from Canberra populations of *C. albitextura* and *C. tenuitela* for which only females were observed. If future research confirms that this second species is thelytokous then it may constitute a heteronomous species that lost its hyperparasitoid male phenotype due to a switch to thelytoky. In contrast to all other parasitoids, H demonstrated little divergence between populations, and appeared to be a single species. Interestingly, this supported an observation in a pollinating fig wasp – parasitoid system that species with functional roles at higher trophic levels are genetically less diverged than their host [69].

Host specialisation and geographic isolation have recently been reported as the main drivers of diversification, resulting in potential cryptic species in braconid wasps of the subfamily Aphidiinae (Hymenoptera: Braconidae) [21]. Furthermore, the generalist strategy of parasitism has also been challenged by the finding of host-specific cryptic species in tachinid flies [22]. A recent study of *Psyllaephagus* parasitoids of three *Mycopsylla* species in Australia also demonstrated a high level of host specificity across their distribution [70]. We therefore conclude host specialisation is a major driver in parasitoid speciation.

All parasitoid morphospecies in our study were either koinobiont endoparasitoids or endohyperparasitoids, which should have greater host specificity because they are constrained by their intimate physiological relationship with their hosts [7]. Diversification processes of P1 were unclear; three putative cryptic species (one per host) were present in three co-occurring Sydney populations of *Cardiaspina* species (*C. maniformis*, *C. vittaformis* and GB *Cardiaspina* sp.), and the P1 of *C. vittaformis* from Sydney was shared by populations of *C. albitextura* and *C. tenuitela* from Canberra and a population of *C. densitexta* from Keith south east of Adelaide. Therefore, phylogenetic congruence analyses did not support codivergence between the P1 morphospecies and its psyllid hosts. TreeMap found evidence of up to four host switch events; however, as the parasitoid phylogeny was not well resolved, it was not possible to determine where these might have occurred. Interestingly, GB *Cardiaspina* sp. did not share P1 with its closest *Cardiaspina* relatives (*C. densitexta* in Keith and *C. tenuitela* in Canberra), while a population of another species from Sydney, *C. vittaformis* shared P1 with *C. densitexta* and *C. tenuitela*.

In contrast, phylogenetic congruence was supported between psyllid hosts and P2 by ParaFit and HCT, although not by TreeMap. A similar result was obtained for *Anicetus* (Encyrtidae) parasitoids and their scale insect hosts, with the conclusion that diversification was driven by sorting events and high host specificity [10] (as was also found between psyllid hosts and P2). The presence of a number of highly host specific cryptic species rather than a single generalist morphospecies appeared likely for P2: populations of the two closely related host species, *C. densitexta* from Keith and GB *Cardiaspina* sp. from Sydney shared the same P2. The more diverged *C. albitextura* from Canberra also shared this P2 genotype, which might be explained by sorting/loss event, or by a host switch. The cophylogenetic analysis of the P2-specific hyperparasitoid *Co. psyllae* (H) demonstrated that the shared P2 between *C. albitextura, C. densitexta* and *C. tenuitela* was the result of a host switch, and this could have occurred as a consequence of the geographic co-occurrence of *C. albitextura* and *C. tenuitela* (albeit we have only detected but not sequenced P2 from *C. tenuitela*).

We then investigated codivergence between the hyperparasitoid *Co. psyllae* (H) and both its primary parasitoid host (P2) and the psyllid host of the primary parasitoid. Species delimitation of *cytb* suggested that H was more generalist on several cryptic species of P2. However, 28S rDNA was more conserved and suggested a single P2 species, although this was not well supported. Despite being an apparent generalist hyperparasitoid species, H was codiverging with its multiple P2 host species based on all three tests of phylogenetic congruence, suggesting possible ongoing speciation of H into host specific species. This is indicative of a long-term association, possibly since its switch from the original host of parasitoids of the genus *Coccidoctonus*, scale insects [71]. Inclusion of a broader host range of *Co. psyllae* and phylogenetic analysis including *Coccidoctonus* species from other hemipteran systems – it also occurs in mealybugs [72] – should provide greater evolutionary resolution of this significant genus of hyperparasitoids.

HH provided an intriguing opportunity to investigate codivergence of a species with hosts across two trophic levels (herbivore and primary parasitoid). We found that HH was codiverging with P1 according to ParaFit, while this was borderline non-significant according to TreeMap and HCT. Sampling from a broader host range may have given a clearer result. However, this trend towards codivergence as a hyperparasitoid of P1 was stronger than its relationship as primary parasitoid with psyllid hosts, for which codivergence was not supported. Host switches of HH occurred between psyllid populations from the same location; *C. albitextura* and *C. tenuitela* from Canberra, and *C. maniformis* and GB *Cardiaspina* sp. from Sydney. HH diversification appeared more strongly driven by geographic differentiation than host specialisation on psyllids; the HH from *C. albitextura* and *C. tenuitela* from Canberra belonged to the same species and genetically diverged from the HH species of four psyllid species from Sydney (where it emerged from *C. fiscella*, *C. maniformis*, GB *Cardiaspina* sp. and *Spondyliaspis* sp.). The weak support for codivergence of HH with P1 may have been influenced by the co-occurring *Cardiaspina* species in Canberra, where they shared P1 and HH genotypes.

## Conclusions

Parasitoid communities in *Cardiaspina* psyllids are dominated by *Psyllaephagus* spp. and *Co. psyllae*. There were many cryptic species in *Psyllaephagus*, and the diversity of this genus may be much higher than currently estimated. Diversification appears to be driven by both host specialisation and host switching between co-occurring hosts. The high host specificity detected for most parasitoid species in this group means that any biological control program will require highly specific parasitoid populations for target psyllids. On the other hand the availability of more generalist parasitoid species may help substitute host specific parasitoids that may not always be available for biological control. From a population dynamic perspective, shared species within parasitoid communities of co-occurring psyllid hosts may also have ecological implications, whereby population fluctuations due to an outbreak of one psyllid species may impact the population dynamics of another psyllid species. The genera *Psyllaephagus* and *Coccidoctonus* may be model genera to study host specificity, diversification and speciation, as well as provide insights into the evolution of hyperparasitism, heteronomous hyperparasitism and thelytoky in parasitoids.

## Additional file

Additional file 1:
**Tables S1 to S8.** (DOCX 54 kb)
