# Supplementary material for: Unravelling mummies: cryptic diversity, host specificity, trophic and coevolutionary interactions in psyllid – parasitoid food webs
Source: BMC Evol Biol. 2017 Jun 6;17:127. doi: 10.1186/s12862-017-0959-2 (PMC5461677; doi:10.1186/s12862-017-0959-2)
Supplement: Additional file 1: — Tables S1 to S8. (DOCX 54 kb) [file 12862_2017_959_MOESM1_ESM.docx]

**Additional file 1:**

**Table S1.** Collection information for psyllid host species from *Eucalyptus*. Collectors were Aidan Hall (AH), Markus Riegler (MR), Martin Steinbauer (MS), Kevin Farnier (KF) and Umar Lubanga (UL).

| **Host species** | **Location** | **Lat/Long** | **Date** | **Collector** | **Host Plant** | |
| --- | --- | --- | --- | --- | --- | --- |
|  |  |  |  |  | **Scientific** | **Common** |
| GB *Cardiaspina* sp. | Koala food plantation, Richmond, NSW | 33°36’S, 150°45’E | May 2014 | AH | *E. moluccana* | Grey Box |
| *C. albitextura* | Alivio Tourist Park, Canberra, ACT | 35°15' S, 149°6' E | April 2014 | MR | *E. blakelyi* | Blakely's Red Gum |
| *C. densitexta* | Mount Monster, Keith, SA | 36°12' S, 140°20' E | October 2013 | MS, KF, UL | *E. fasciculosa* | Pink Gum |
| *C. fiscella* | EucFACE, Richmond, NSW | 33°37' S, 150°44' E | September 2014 | AH, MR | *E. tereticornis* | Forest Red Gum |
| *C. maniformis* | Mary St, Macquarie Fields, NSW | 33°59' S, 150°53' E | September 2014 | AH | *E. tereticornis* | Forest Red Gum |
| *C. tenuitela* | Alivio Tourist Park, Canberra, ACT | 35°15' S, 149°6' E | April 2014 | MR | *E. melliodora* | Yellow Box |
| *C. vittaformis* | Bungarribee Rd, Blacktown, NSW | 33°46' S, 150°52' E | September 2014 | AH | *E. crebra* | Narrow-leaved Ironbark |
| **Outgroup** |  |  |  |  |  |  |
| *Spondyliaspis* sp. | EucFACE, Richmond, NSW | 33°37' S, 150°44' E | September 2014 | AH, MR | *E. tereticornis* | Forest Red Gum |

**Table S2.** Voucher numbers of parasitoid morphotypes at the Australian National Insect Collection (CSIRO, Canberra) from two of the *Cardiaspina* spp. hosts, collected when their psyllid hosts were at significant outbreak levels in the Cumberland Plain Woodland of Western Sydney. Parasitoid morphotypes were designated the abbreviations P1 and P2 (primary parasitoids), H (hyperparasitoid) and HH (heteronomous hyperparasitoid).

| ***Cardiaspina* host** | **Parasitoid morphotype** | **ANIC**  **database number** |
| --- | --- | --- |
| *C. fiscella* | P1 female | 32-069126 |
|  | P2 male | 32-069121 |
|  | P2 female | 32-069120 |
|  | H male | 32-069103 – 32-069112 |
|  | H female | 32-069113 – 32-069119 |
|  | HH male | 32-069122 – 32-069123 |
|  | HH female | 32-069124 – 32-069125 |
| GB *Cardiaspina* sp. | P1 female | 32-069048 – 32-069051 |
|  | P2 male | 32-069080 – 32-069094 |
|  | P2 female | 32-069095 – 32-069102 |
|  | H male | 32-069052 – 32-069063 |
|  | H female | 32-069064 – 32-069079 |
|  | HH male | 32-069041 – 32-069047 |
|  | HH female | 32-069033 – 32-069040 |

**Table S3.** GenBank accession numbers for *cytb* and 28S rDNA of the encyrtid parasitoid morphospecies obtained from *Cardiaspina* and *Spondyliaspis* sp. host populations. Individual number corresponds to the same individual for both genetic markers, and the genotype number corresponds to the assigned concatenated genotype. P1, P2 and HH are *Psyllaephagus* species, H is *Coccidoctonus psyllae*.

| **Host species** | **Parasitoid morphotype** | ***cytb* Genbank accession number** | **Individual number** | **28S rDNA Genbank accession number** | **Individual number** | **Genotype number** |
| --- | --- | --- | --- | --- | --- | --- |
| *C. albitextura* | P1 | KU568376 | 1 - 4 | KU568423 | 1 - 4 | GT1 |
|  | P2 | KU568377 KU568378 | 1 - 3 | KU568424 | 1 - 4 | GT1 GT2 |
|  |  |  | 4 |  |  |  |
|  | H | KU568379 KU568380 KU568381 KU568382 | 1 | KU568425 | 1 - 4 | GT1 GT2 GT3 GT4 |
|  |  |  | 2 |  |  |  |
|  |  |  | 3 |  |  |  |
|  |  |  | 4 |  |  |  |
|  | HH | KU568383 KU568384 | 1 - 2 | KU568426 | 1 - 4 | GT1 GT2 |
|  |  |  | 3- 4 |  |  |  |
| *C. densitexta* | P1 | KU568385 KU568386 | 1 | KU568427 | 1 - 4 | GT1 GT2 |
|  |  |  | 2 - 4 |  |  |  |
|  | P2 | KU568387 | 1 - 4 | KU568428 | 1 - 4 | GT1 |
|  | H | KU568388 KU568389 KU568390 | 1 | KU568429 | 1 - 4 | GT1 GT2 GT3 GT4 |
|  |  |  | 2 - 3 |  |  |  |
|  |  |  | 4 |  |  |  |
| *C. fiscella* | P2 | KU568391 KU568392 | 1 - 2 | KU568430 | 1 - 4 | GT1 GT2 |
|  |  |  | 3 - 4 |  |  |  |
|  | H | KU568393 | 1 - 4 | KU568431 | 1 - 4 | GT1 |
|  | HH | KU568394 | 1 - 4 | KU568432 KU568433 | 1, 3 - 4 2 | GT1 GT2 |
|  |  |  |  |  |  |  |
| *C. maniformis* | P1 | KU568395 | 1 - 4 | KU568434 | 1 - 4 | GT1 |
|  | P2 | KU568396 | 1 - 4 | KU568435 | 1 - 4 | GT1 |
|  | HH | KU568397 KU568398 KU568399 | 1 - 2 | KU568436 KU568437 | 1, 4 2 - 3 | GT1 GT2 GT3 GT4 |
|  |  |  | 3 |  |  |  |
|  |  |  | 4 |  |  |  |
| *C. tenuitela* | P1 | KU568400 | 1 - 4 | KU568438 | 1 - 4 | GT1 |
|  | H | KU568401 KU568402 KU568403 | 1 - 2 | KU568439 | 1 - 4 | GT1 GT2 GT3 GT4 |
|  |  |  | 3 |  |  |  |
|  |  |  | 4 |  |  |  |
|  | HH | KU568404 KU568405 | 1 | KU568440 | 1 - 4 | GT1 GT2 |
|  |  |  | 1 - 3 |  |  |  |
| *C. vittaformis* | P1 | KU568406 KU568407 KU568408 | 1 - 2 | KU568441 | 1 - 4 | GT1 GT2 GT3 GT4 |
|  |  |  | 3 |  |  |  |
|  |  |  | 4 |  |  |  |
| GB *Cardiaspina* sp. | P1 | KU568409 KU568410 KU568411 | 1 - 2 | KU568442 KU568443 | 1 2 - 4 | GT1 GT2 GT3 GT4 |
|  |  |  | 3 |  |  |  |
|  |  |  | 4 |  |  |  |
|  | P2 | KU568412 KU568413 | 1 - 2 | KU568444 | 1 - 4 | GT1 GT2 |
|  |  |  | 3 - 4 |  |  |  |
|  | H | KU568414 KU568415 KU568416 | 1 | KU568445 KU568446 | 1 - 2, 4 3 | GT1 GT2 GT3 GT4 |
|  |  |  | 2 |  |  |  |
|  |  |  | 3 - 4 |  |  |  |
|  | HH | KU568417 | 1 - 4 | KU568447 | 1 - 4 | GT1 |
| *Spondyliaspis* sp. | P2 | KU568418 KU568419 KU568420 | 1 - 2 | KU568448 | 1 - 4 | GT1 GT2 GT3 GT4 |
|  |  |  | 3 |  |  |  |
|  |  |  | 4 |  |  |  |
|  | H | KU568421 | 1 - 3 | KU568449 | 1 - 3 | GT1 |
|  | HH | KU568422 | 1 - 4 | KU568450 | 1 - 4 | GT1 |
|  | aphelinid sp. | not obtained |  | KU568451 | 1 - 2 | not applicable |

**Table S4.** List of primers and PCR conditions used in this study to construct the psyllid phylogeny. Nuclear primers were designed in the program PriFi based on sequence alignments of *wg, EF-1 alpha* and *CAD* genes of *C. albitextura, C. tenuitela, C. densitexta* and GB *Cardiaspina* sp. obtained from Illumina HiSeq total genomic shotgun sequences of single adult males searched by using GenBank sequence information of these genes from a range of hemipteran insects (Morrow *et al*. unpublished data).

| **Primer** | **Target gene** | **Size (bp)** | **Primer sequence (5' - 3')** | **PCR cycling conditions** | **Evolutionary model** | **Reference** |
| --- | --- | --- | --- | --- | --- | --- |
| Dick Pat | *COI* | 506 | CCAACAGGAATTAAAATTTTTAGATGATTAGC TCCAATGCACTAATCTGCCATATTA | 94 °C for 3 minutes; 35 cycles of 94 °C for 30 seconds, 50 °C for 30 seconds and 72 °C for 1 minute; 72 °C for 10 minutes | HKY + G | Simon *et al*. 1994 |
| CB1 CB2 | *cytb* | 398 | TATGTACTACCATGAGGACAAATATC ATTACACCTCCTAATTTAATTAGGAAT | 94 °C for 3 minutes; 30 cycles of 95 °C for 15 seconds, 45 °C for 20 seconds and 72 °C for 1 minute; 72 °C for 10 minutes | HKY + G | Jermiin & Crozier 1994 |
| PswgF PswgR | *wg* | 268 | ACATGYTGGATGAGAYTACCA TCTTGTGTTCTATAACCACGCCCAC | 94 °C for 3 minutes; 30 cycles of 94 °C for 30 seconds, 58 °C for 30 seconds and 72 °C for 30 seconds; 72 °C for 10 minutes | K2 + G | This study |
| PsEF1aF PsEF1aR | *EF-1 alpha* | 281 | CAGTACCTGTTGGTCGTGTTGAGAC ACGACGRTCACAYTTTTCTTTGATC | 94 °C for 3 minutes; 35 cycles of 94 °C for 30 seconds, 50 °C for 30 seconds and 72 °C for 1 minute; 72 °C for 10 minutes | K2 | This study |
| PsCADF PsCADR | *CAD* | 323 | CGTATGGTAGATGAAAGTGT AATTTGTTTGWGCAGGATAYTCTGC | 94 °C for 3 minutes; 35 cycles of 94 °C for 30 seconds, 50 °C for 30 seconds and 72 °C for 1 minute; 72 °C for 10 minutes | T92 + G | This study |

**Table S5.** GenBank accession numbers for psyllid DNA sequences.

| **Psyllid species** | ***COI*** | ***cytb*** | ***CAD*** | ***EF-1 alpha*** | ***wg*** |
| --- | --- | --- | --- | --- | --- |
| *C. albitextura* | KU568244 | KU568258 | KU568231 | KU568272 | KU568348 |
| *C. densitexta* | KU568250 | KU568264 | KU568237 | KU568278 | KU568354 |
| *C. fiscella* | KU568253 | KU568267 | KU568240 | KU568281 | KU568357 |
| *C. maniformis* | KU568248 | KU568262 | KU568235 | KU568276 | KU568352 |
| *C. tenuitela* | KU568247 | KU568261 | KU568234 | KU568275 | KU568351 |
| *C. vittaformis* | KU568251 | KU568265 | KU568238 | KU568279 | KU568355 |
| GB *Cardiaspina* sp. | KU568249 | KU568263 | KU568236 | KU568277 | KU568353 |

**Table S6.** Morphospecies-specific mitochondrial cytochrome b primers designed for the parasitoid species associated with five *Cardiaspina* spp. and one *Spondyliaspis* sp. host populations. Primers for parasitoids from *C. densitexta* and *C. vittaformis* were not developed because no post-emergence mummies were sampled from these two species.

| **Target species** | **Biology** | **Host population** | **Primer** | **Target gene** | **Primer sequence (5' - 3')** | **Amplicon**  **size (bp)** |
| --- | --- | --- | --- | --- | --- | --- |
| P1a *Psyllaephagus* sp. | Primary parasitoid | *C. fiscella C. maniformis* GB *Cardiaspina* sp. *Spondyliaspis* sp. | P1aCBfor P1aCBrev | cytochrome b | AGCAATCCCTTACTTAGGAAATACAG GAACGTAAAATTGCATAAGCAAAAAG | 400 |
| P1b *Psyllaephagus* sp. | Primary parasitoid | *C. albitextura C. tenuitela* | P1bCBfor P1bCBrev | cytochrome b | ATTAATAATGCAACCCTTAATCGATTCTAC CGTAAAATCGCGTAAGCAAATAGG | 340 |
| P2 *Psyllaephagus* sp. | Primary parasitoid | All | P2CBfor P2CBrev | cytochrome b | GATTTTATTCATTTCATTTCATTTTACCA TATGAGTATGATGTACAAATCAATAAAAGT | 215 |
| H *Coccidoctonus psyllae* | Hyperparasitoid | All | HCBfor HCBrev | cytochrome b | TTGTACTATGATTATGAGGAGGGTTC GGAGTCACTATTGGGTTAGCTTTA | 315 |
| HHa *Psyllaephagus* sp. | Heteronomous hyperparasitoid | *C. fiscella C. maniformis* GB *Cardiaspina* sp. *Spondyliaspis* sp. | HHaCBfor HHaCBrev | cytochrome b | GGAGATAATATTGTTTTATGACTATGAGGT CCCRGTTTCATGAAGAAATATTAAATGG | 145 |
| HHb *Psyllaephagus* sp. | Heteronomous hyperparasitoid | *C. albitextura C. tenuitela* | HHbCBfor HHbCBrev | cytochrome b | TTTATTCATTTCATTTTATTATACCTTTTG AATAAACTGTGCATACAATTAAAAGG | 205 |
| *Cardiaspina* sp. psyllid | Host | All | wg72F wg361R | *wingless* | GACGGTGCTTCTAGAGTAATGG GCACATCGTTCCACAACAATAA | 235 |

**Table S7.** Sequence divergence (%) comparisons of 28S rDNA (509 bp) between populations of parasitoids from their associated psyllid hosts.

| **Comparison** | **P1** | **P2** | **H** | **HH** |
| --- | --- | --- | --- | --- |
| *C. albitextura - C. densitexta* | 0 | 0 | 0 | – |
| *C. albitextura - C. fiscella* | – | 1.7 | 1.9 | 6.3 |
| *C. albitextura - C. maniformis* | 8.2 | 0.6 | – | 4.4 |
| *C. albitextura - C. tenuitela* | 0 | – | 0 | 0 |
| *C. albitextura - C. vittaformis* | 3 | – | – | – |
| *C. albitextura -* GB *Cardiaspina* sp. | 8.4 | 0.2 | 0.4 | 3.8 |
| *C. albitextura - Spondyliaspis* sp. | – | 1.5 | 0.8 | 3.8 |
| *C. densitexta - C. fiscella* | – | 1.7 | 1.9 | – |
| *C. densitexta - C. maniformis* | 8.2 | 0.6 | – | – |
| *C. densitexta - C. tenuitela* | 0 | – | 0 | – |
| *C. densitexta - C. vittaformis* | 3 | – | – | – |
| *C. densitexta -* GB *Cardiaspina* sp. | 8.4 | 0.2 | 0.4 | – |
| *C. densitexta - Spondyliaspis* sp. | – | 1.5 | 0.8 | – |
| *C. fiscella - C. maniformis* | – | 1.1 | – | 2.5 |
| *C. fiscella - C. tenuitela* | – | – | 1.9 | 6.3 |
| *C. fiscella - C. vittaformis* | – | – | – | – |
| *C. fiscella -* GB *Cardiaspina* sp. | – | 1.5 | 1.9 | 2.5 |
| *C. fiscella - Spondyliaspis* sp. | – | 2.3 | 1.5 | 2.5 |
| *C. maniformis - C. tenuitela* | 8.2 | – | – | 4.4 |
| *C. maniformis - C. vittaformis* | 10.6 | – | – | – |
| *C. maniformis -* GB *Cardiaspina* sp. | 3 | 0.4 | – | 0.6 |
| *C. maniformis - Spondyliaspis* sp. | – | 1.3 | – | 0.6 |
| *C. tenuitela - C. vittaformis* | 3 | – | – | – |
| *C. tenuitela -* GB *Cardiaspina* sp. | 8.4 | – | 0.4 | 3.8 |
| *C. tenuitela - Spondyliaspis* sp. | – | – | 0.8 | 3.8 |
| *C. vittaformis -* GB *Cardiaspina* sp. | 10.6 | – | – | – |
| *C. vittaformis - Spondyliaspis* sp. | – | – | – | – |
| GB *Cardiaspina* sp. *- Spondyliaspis* sp. | – | 1.3 | 0.8 | 0 |

**Table S8.** bPTP putative species delimitation analysis of 28S rDNA from parasitoid morphospecies from different host populations.

| **Parasitoid morphospecies** | **bPTP putative species populations** | **Posterior delimitation probability** |
| --- | --- | --- |
| P1 | GB *Cardiaspina* sp. | 0.81 |
| P1 | *C. maniformis* | 0.95 |
| P1 | *C. albitextura*, *C. densitexta*, *C. tenuitela*, *C. vittaformis* | 0.93 |
| P2 | *C. albitextura*, *C. densitexta*, *C. fiscella*, *C. maniformis*, GB *Cardiaspina* sp., *Spondyliaspis* sp. | 0.57 |
| H | *C. albitextura*, *C. densitexta*, *C. fiscella*, *C. tenuitela*, GB *Cardiaspina* sp., *Spondyliaspis* sp. | 0.72 |
| HH | *C. fiscella*, *C. maniformis*, GB *Cardiaspina* sp., *Spondyliaspis* sp. | 0.31 |
| HH | *C. albitextura*, *C. tenuitela* | 0.96 |
